# Editorial: Problem Gambling: Summarizing Research Findings and Defining New Horizons

**DOI:** 10.3389/fpsyg.2018.01670

**Published:** 2018-09-06

**Authors:** Tobias Hayer, Caterina Primi, Neven Ricijas, Daniel T. Olason, Jeffrey L. Derevensky

**Affiliations:** ^1^Institute of Psychology and Cognition Research, University of Bremen, Bremen, Germany; ^2^Department of Neurofarba, University of Florence, Florence, Italy; ^3^Faculty of Education and Rehabilitation Sciences, University of Zagreb, Zagreb, Croatia; ^4^University of Iceland, Reykjavik, Iceland; ^5^McGill University, Montreal, QC, Canada

**Keywords:** gambling, problem gambling, adolescence, measurement, risk factors, prevention, treatment

## Introduction

More than a decade ago, Shaffer et al. (2006) reported that gambling-related research was growing at an exponential rate. Since that time, this trend appears to have continued, and much more is now known about this particular form of risky behavior. Nevertheless, there is still a general tendency to not perceive gambling as a potential danger for youth and other vulnerable populations.

The latest edition of the Diagnostic and Statistical Manual of Mental Disorders (DSM-5) included “gambling disorder” as the only condition in the section “non-substance-related disorders.” Moreover, it was specified that this disorder can indeed occur in adolescence, young adulthood or even late adulthood. Despite this fact, theoretical and applied research on problem gambling especially with regard to adolescence and other risk groups still remains fragmentary. For this reason, we felt it to be important to organize a special research topic on gambling. The primary goals were to highlight the necessity of considering excessive gambling as a potential harmful activity, to summarize the state-of-art of international research on different aspects of the topic and to offer important novel findings relevant for advancing knowledge in the field of gambling. Taken together, the contributions can be classified into four broad categories: (1) youth gambling, (2) risk factors in adulthood, (3) measurement issues, and (4) clinical research.

## Overview of contributing papers

In total, 18 papers are presented in this special issue. The first central domain refers to gambling among youth. Even though regulated forms of gambling are generally prohibited to minors, there is a considerable body of research that proves their involvement in gambling activities. A significant minority of adolescents even show gambling-related psychosocial problems (Calado et al., 2017). In addition, several studies have explored risk and protective factors in childhood, adolescence or young adulthood for the development of problem gambling symptoms (Dowling et al., 2017). Four papers in this issue have specifically focused on youth gambling, contributing to the current knowledge by exploring less studied psychosocial constructs or subpopulations and offering guidelines for the conception of interventions. From the broader social perspective, Canale et al. presented the first study with a large-scale nationally representative sample of adolescents to examine the effects of income inequality on adolescent gambling, concluding that wealth distribution may have an impact on youth gambling. Gender issues were raised with the study from Huic et al. focusing on gambling predictors of adolescent girls who are a much less studied population than boys. Furthermore, empirical findings from Nigro et al. with regard to different emotional and cognitive factors confirmed the impact of impulsivity and emotional distress on the development of youth problem gambling. Last but not least, Donati et al. addressed mindware problems (i.e., cognitive distortions) and their influence both on youth gambling as well as the conception of theoretically founded preventive interventions.

In addition, four papers shed light on specific risk constellations for the development and manifestation of gambling-related problems in adulthood. Based on representative data from Austria, Buth et al. tackled the question of whether certain risk factors are equally relevant for at-risk, problem, and disordered gamblers. Overall, their findings indicated that the included risk factors indeed differ between these gambling groups, suggesting the need for more tailored prevention and treatment strategies. In contrast to this approach, the study by Hing et al. aimed at identifying risk factors for three forms of problematic online gambling [i.e., electronic gaming machines (EGMs), sports betting, race betting]. While the risk profiles of online sports bettors and race bettors were largely similar, a rather different pattern emerged for online EGM gamblers pointing again to the importance of differential activities in terms of prevention and intervention. Unique findings also stem from Olason et al. who conducted a population-based follow-up study in order to determine the impact of the economic crisis in Iceland on gambling behavior. Interestingly, past year problematic gambling figures did not change after the economic collapse. However, an increased participation in lotto and scratch tickets indicates that gambling forms with low initial stakes and large jackpots may then become more enticing, in particular for individuals suffering financial difficulties. In a very well-balanced opinion paper Zakiniaeiz et al. finally recalled the necessity to study gender differences in gambling patterns, especially with regard to preferred gambling forms, the onset of disordered gambling, co-occurring disorders and disorder progression.

Another important area in gambling research relates to measurement issues. In particular, the reliable and valid assessment of problem gambling patterns has received a considerable amount of attention for both adolescents (Edgren et al., 2016) and adults (Pickering et al., 2018). Five papers deal with the psychometric properties of novel measurement tools. Against the background that large-scale prevalence studies consistently represent high prevalence rates of gambling participation among youth (see above), two papers directly focus on this age cohort. While Stinchfield et al. developed and evaluated the psychometric properties of the Brief Adolescent Gambling Screen (BAGS), a three-item screen for adolescent problem gambling, Donati et al. tested the gender invariance of their Gambling Behavior Scale for Adolescents (GBS-A) applying item response theory. New tools that broadly aim at determining risk and protective factors associated with problem gambling in adults were also introduced. For example, Barbaranelli et al. reported the psychometric properties of the Multidimensional Gambling Self-Efficacy Scale (MGSES), an innovative scale to measure self-efficacy as a protective factor for problem gambling. In addition, Cowie et al. provided preliminary evidence for the predictive validity of the Gambling Cognitions Inventory (GCI) as a measure of cognitive distortions, showing its relationship to several gambling outcomes over a 1-month and a 6-month time period, respectively. In a similar vein, Jonsson et al. assessed the capacity of the different dimensions of the Jonsson-Abbott Scale (JAS) to predict increases in problem gambling risk levels as well as the onset of problem gambling over 1 year.

The final main subject of interest relates to clinical examinations of problem gambling. Researchers and treatment providers have sought to identify the underlying issues associated with problem gambling and have tried to identify both the barriers preventing individuals for seeking help and best practices in working with individuals with this disorder. Five informative papers have looked at this issue from multiple perspectives. Challet-Bouju et al. provided a systematic review of cognitive interventions highlighting that this common form of intervention represents a promising approach to gambling disorder management while Tremblay et al. documented the experiences of gamblers and their partners either individually or in couple therapy. Their conclusion was that both forms of treatment were effective but more positive experiences emerged for couple therapy. In yet another interesting paper, Gavriel-Fried and Rabayov examined the importance of self-stigma for individuals seeking treatment for gambling, alcohol or other substance use problems. They summarized that stigma among individuals with gambling problems tend to work in a similar way as among those individuals with an alcohol or drug problem. Jiménez-Murcia et al. analyzed the frequency of the co-occurrence of gambling disorders and food addiction. Their findings suggest that almost 10% of individuals having a gambling disorder concurrently experienced a food addiction. In addition, a far higher ratio of food addiction was found in women. Lastly, Giroux et al. provided a systematic review of online and mobile interventions for problem gambling, alcohol and drug use. While this may prove promising in the future, more rigorous research is necessary before definite conclusions can be reached. In sum, more research is clearly needed in understanding gambling disorders or problem gambling patterns before best practice treatment approaches can be identified. Clinicians and treatment providers are well aware that problem gamblers do not represent a homogenous group (Blaszczynski and Nower, 2002) and that differential approaches may be required.

Overall, 94 different authors from 15 countries contributed to this special issue. We remain confident that these 18 papers significantly add to the understanding of problem gambling and will further stimulate high-quality gambling research in its many facets.

## Author contributions

All authors listed have made a substantial, direct and intellectual contribution to the work, and approved it for publication.

### Conflict of interest statement

The authors declare that the research was conducted in the absence of any commercial or financial relationships that could be construed as a potential conflict of interest.
